# CT findings of glycogen storage disease I complicated with pancreatitis: A case report

**DOI:** 10.1097/MD.0000000000033668

**Published:** 2023-04-28

**Authors:** Shuo Chen, Feng Chen, Fei Cheng, Xiao Xu

**Affiliations:** a Department of Radiology, First Affiliated Hospital, Zhejiang University School of Medicine, Hangzhou, Zhejiang Province, China; b Department of Pathology, First Affiliated Hospital, Zhejiang University School of Medicine, Hangzhou, Zhejiang Province, China; c Department of Radiology, Shaoxing People’s Hospital (Shaoxing Hospital, Zhejiang University School of Medicine), Shaoxing, China; d Key Laboratory of Functional Molecular Imaging of Tumor and Interventional Diagnosis and Treatment of Shaoxing City, Shaoxing, China.

**Keywords:** computed tomography, glycogen storage disease type I, pancreatitis, X-ray

## Abstract

**Rationale:**

The incidence of glycogen storage disease type I (GSD I) in the overall population is 1/100,000.^[1]^ Hyperlipidemia in patients with GSD I can induce pancreatitis. Three cases of GSD I complicated with pancreatitis have been reported.^[2]^ Here, the computed tomography (CT) features of GSD I complicated with pancreatitis are reported for the first time

**Patient concerns:**

A 22-year-old female presents with growth retardation for 20 years and recurrent epigastric pain for 3 years. No abnormality in physical examination. Laboratory examination: GPT 81 U/L, GOT 111 U/L, DBIL 1.7 umol/L, TBIL 0.7 umol/L, Albumen 41.4 g/L, blood ammonia 54 umol/L, fasting blood glucose 3.02 mmol/L, G6PD 1829 U/L, lactic acid 7.9 mmol/L, triglyceride 18.79 mmol/L, TCH 9.46 mmol/L, uric acid 510 umol/L, urinary protein +++ (3.0) g/L.

**Diagnosis:**

The CT findings of the upper abdomen show that the liver is obviously enlarged, and the density of the liver is obviously uneven on plain scan. Unclear boundaries and increased blood vessels of the pancreas are found, especially in the head of the pancreas. The patient is diagnosed with GSD I complicated with pancreatitis.

**Interventions:**

The patient undergoes split liver transplantation and splenectomy under general anesthesia in our hospital.

**Outcomes:**

Upper abdominal CT is reexamined half a month and 2 and a half months after the operation. It is found that the transplanted liver is not enlarged and the density is not abnormal. The pancreas shrinks, its boundary is clear, and its blood vessels decrease, especially in the head of the pancreas

**Lessons:**

The density of the liver depends on the relative amount of glycogen and fat, which can be increased, normal, or decreased. Hyperlipidemia in patients with GSD I can induce pancreatitis.

## 1. Introduction

A case of glycogen storage disease I complicated with pancreatitis is reported. The computed tomography (CT) features are summarized as follows. The liver is enlarged. There are high-density glycogen storage areas and low-density fat infiltration areas in the plain CT scan of the liver. Unclear boundaries and increased blood vessels in the pancreas are found.

## 2. Case report

A 22-year-old female presents with growth retardation for 20 years and recurrent epigastric pain for 3 years. No abnormality in the physical examination. Laboratory examination: GPT 81 U/L, GOT 111 U/L, DBIL 1.7 umol/L, TBIL 0.7 umol/L, albumen 41.4 g/L, blood ammonia 54 umol/L, fasting blood glucose 3.02 mmol/L, G6PD 1829 U/L, lactic acid 7.9 mmol/L, triglyceride 18.79 mmol/L, TCH 9.46 mmol/L, uric acid 510 umol/L, urinary protein +++ (3.0) g/L.

**Figure 1.**
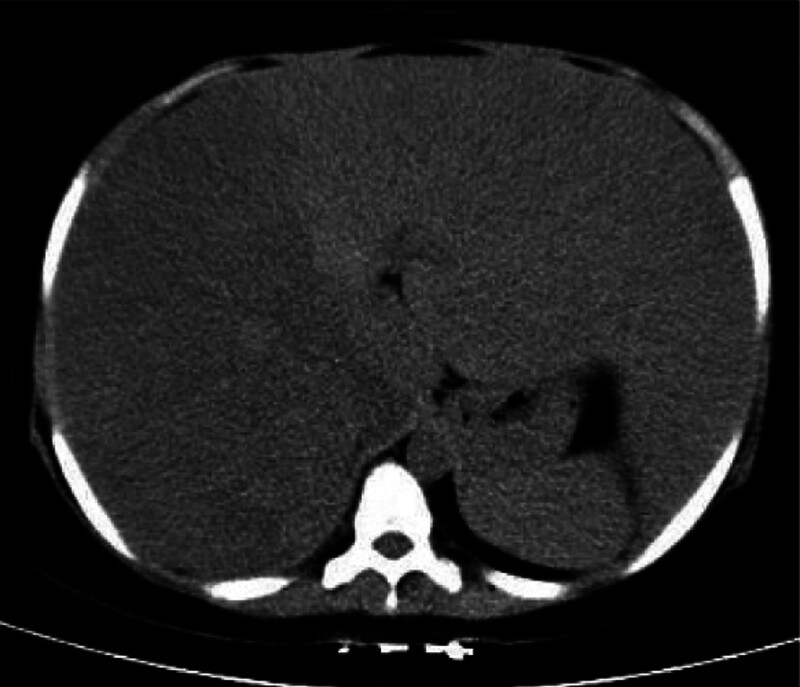
A plain CT scan of the upper abdomen. The liver is obviously enlarged, its density is obviously uneven, and the mosaic-like appearance is seen. The density of the right lobe is slightly lower than that of the left lobe. CT = computed tomography.

**Figure 2.**
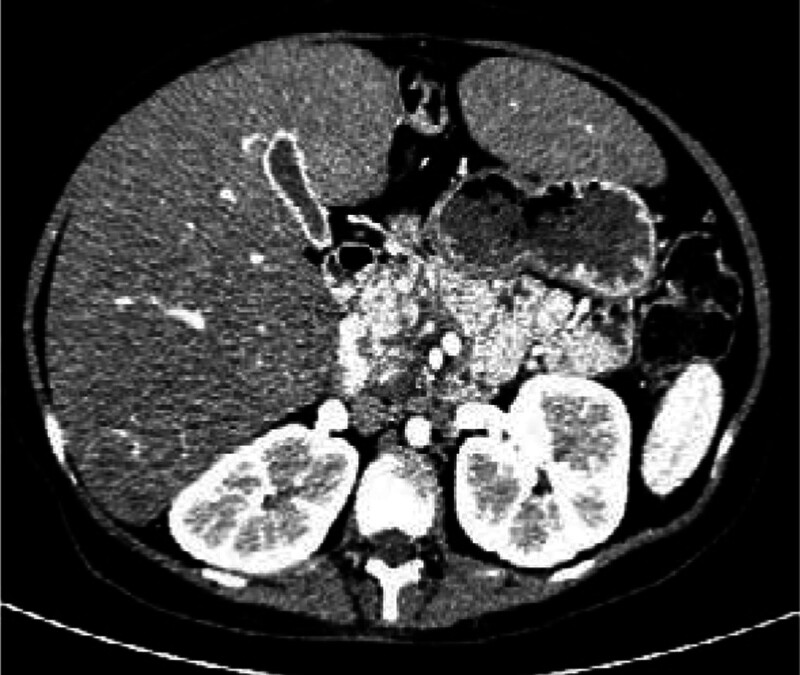
An enhanced CT scan of the upper abdomen. Unclear boundaries and increased blood vessels in the pancreas are found. CT = computed tomography.

The CT findings of the upper abdomen show that the liver is obviously enlarged, the density of the liver is obviously uneven on plain scan, and the density of the right lobe of the liver is slightly lower than that of the left lobe of the liver. The CT value of the right lobe of the liver is between 12 and 69HU and the CT value of the left lobe of the liver is between 24 and 76HU (Fig. 1). The liver is gradually enhanced. Unclear boundaries and increased blood vessels of the pancreas are found, especially in the head of the pancreas (Fig. 2).

**Figure 3.**
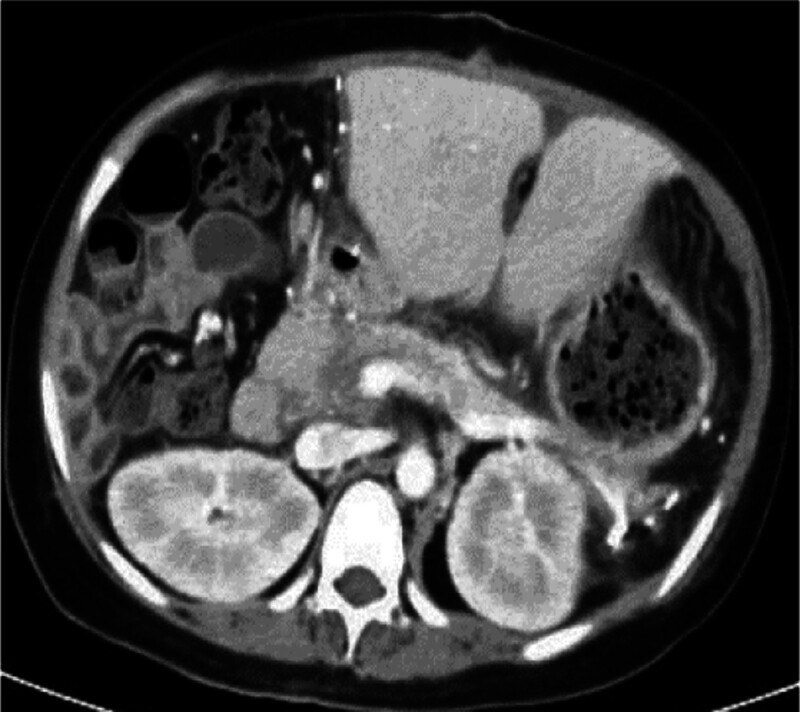
Enhanced CT scans of the upper abdomen half a month and 2 and a half months after the operation, respectively. The pancreas shrinks, its boundary is clear, and its blood vessels decrease, especially in the head of the pancreas. Ischemic infarcted area without enhancement is seen in the head of the pancreas (black arrow). CT = computed tomography.

**Figure 4.**
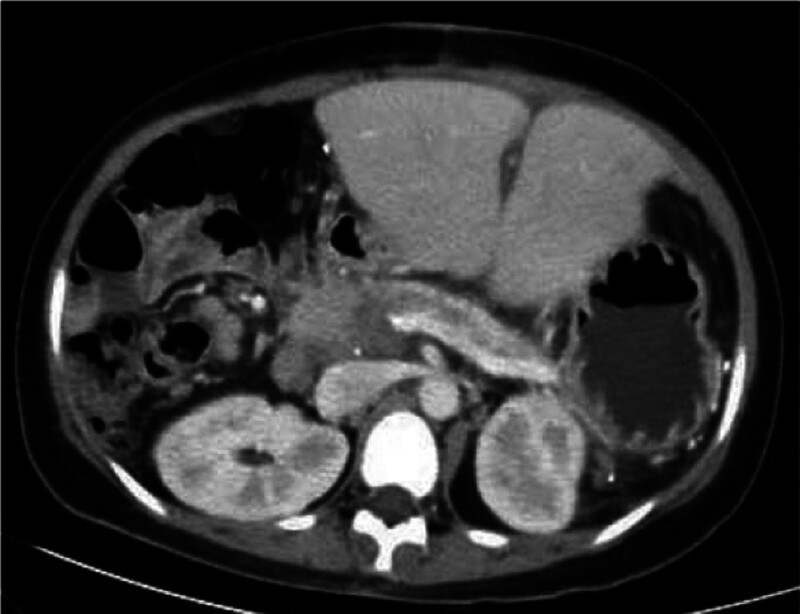
Enhanced CT scans of the upper abdomen half a month and 2 and a half months after the operation, respectively. The pancreas shrinks, its boundary is clear, and its blood vessels decrease, especially in the head of the pancreas. Ischemic infarcted area without enhancement is seen in the head of the pancreas (black arrow). CT = computed tomography.

The patient is diagnosed with glycogen storage disease type I (GSD I) complicated with pancreatitis, who has the indication for liver transplantation^[3]^ and has no specific contraindications for liver transplantation. The patient undergoes split liver transplantation and splenectomy under general anesthesia in our hospital. A small amount of ascites is detected during the operation, and the liver is tough and enlarged in volume. Microscopically, the normal structure of the hepatic lobule is destroyed and a false lobule is formed. The hepatocytes are obviously enlarged and the staining becomes lighter. The hepatocytes are disorderly arranged and the hepatic sinusoids are compressed. Steatosis is seen in hepatocytes. A few inflammatory cells infiltrate the stroma. Fibrous tissue proliferates and forms fibrous septum (Fig. 3). Immunohistochemistry PAS (+) (Fig. 4).

**Figure 5.**
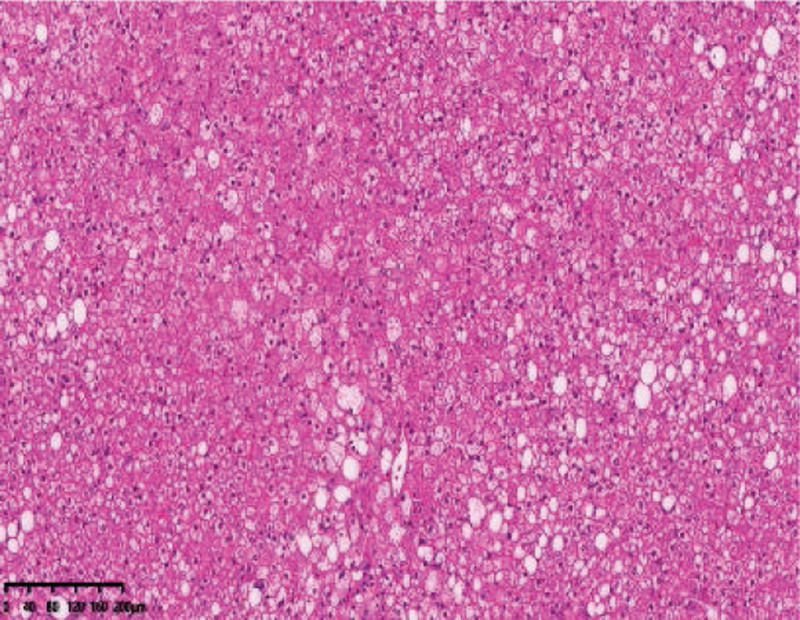
HE staining image (100x) shows that the hepatocytes are enlarged, the cytoplasm is loose, and the organelles are pushed under the cell membrane by a large number of stored glycogen particles. Steatosis and glycogen nucleus can be seen in the liver tissue at the same time, so the appearance of a mosaic can be seen. HE = hematoxylin and eosin.

**Figure 6.**
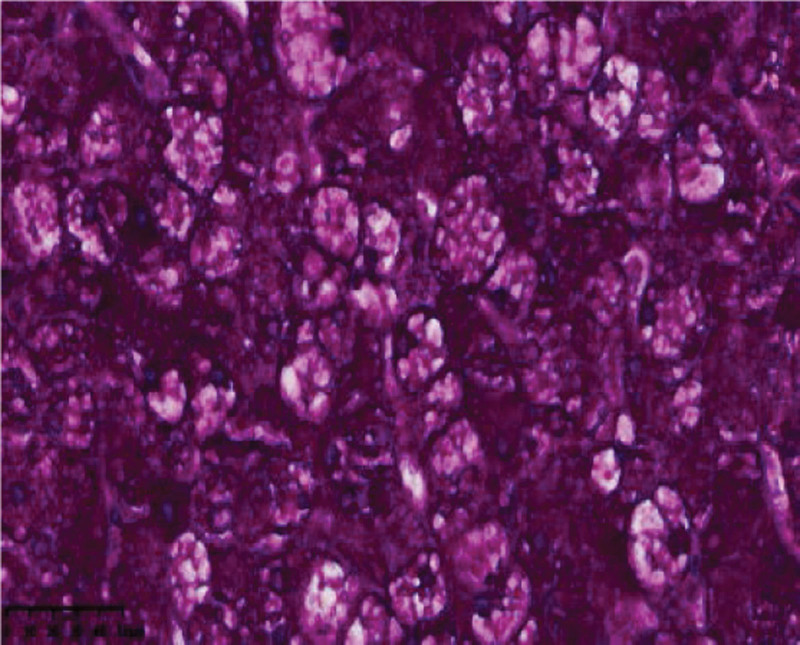
PAS staining image (400x) shows that PAS diffuse cherry red staining is seen in the cytoplasm of hepatocytes, indicating that there are a large number of glycogen granules in hepatocytes.

Upper abdominal CT is reexamined half a month and 2 and a half months after the operation. It is found that the transplanted liver is not enlarged and the density is not abnormal. The pancreas shrinks, its boundary is clear, and its blood vessels decrease, especially in the head of the pancreas. Ischemic infarcted area without enhancement is seen in the head of the pancreas (Figs. 5 and 6).

## 3. Discussion

GSD I is an autosomal recessive inborn error of carbohydrate metabolism caused by defects of the glucose-6-phosphatase complex.^[2]^ Deficiency of glucose-6-phosphatase activity in the liver, kidney, and intestine results in the accumulation of glycogen in these organs.^[2]^ As a result of inadequate glucose production, patients have severe fasting hypoglycemia with secondary biochemical abnormalities: hyperlactacidemia, hyperuricemia, and hyperlipidemia.^[2]^ The incidence of GSD I in the overall population is 1/100,000.^[1]^

Hyperlipidemia in patients with GSD I can induce pancreatitis. Three cases of GSD I complicated with pancreatitis have been reported.^[2]^ Here, the CT features of GSD I complicated with pancreatitis are reported for the first time.

Studies have shown that diffuse or focal high-density areas in the plain CT scan for the liver, are more glycogen deposition areas in the histological examination in patients with GSD I. If there is fat infiltration at the same time, it can completely or partially counteract the effect of glycogen on liver density. The density of the liver depends on the relative amount of glycogen and fat, which can be increased, normal, or decreased.^[4]^

## 4. Conclusion

Here, the CT features of GSD I complicated with pancreatitis are reported for the first time. The liver is enlarged. There are high-density glycogen storage areas and low-density fat infiltration areas in the plain CT scan of the liver. Unclear boundaries and increased blood vessels in the pancreas are found.

## Author contributions

**Data curation:** Shuo Chen, Fei Cheng.

**Resources:** Fei Cheng.

**Supervision:** Feng Chen, Xiao Xu.

**Validation:** Feng Chen.

**Visualization:** Xiao Xu.

**Writing – original draft:** Shuo Chen.

**Writing – review & editing:** Shuo Chen, Feng Chen, Xiao Xu.
